# Effects of self-assessed chewing ability, tooth loss and serum albumin on mortality in 80-year-old individuals: a 20-year follow-up study

**DOI:** 10.1186/s12903-020-01113-7

**Published:** 2020-04-21

**Authors:** Yoshiaki Nomura, Erika Kakuta, Ayako Okada, Ryoko Otsuka, Mieko Shimada, Yasuko Tomizawa, Chieko Taguchi, Kazumune Arikawa, Hideki Daikoku, Tamotsu Sato, Nobuhiro Hanada

**Affiliations:** 1 Department of Translational Research, Tsurumi University School of Dental Medicine, 2-1-3 Tsurumi, Tsurumi-ku, Yokohama, 230-8501 Japan; 2 Department of Oral bacteriology, Tsurumi University School of Dental Medicine, Kanagawa, Japan; 3 Chiba Prefecture University of Health Sciences, Chiba, Japan; 4 Department of Cardiovascular Surgery, Tokyo Women’s Medical University, Tokyo, Japan; 5 Department of Preventive and Public Oral Health, Nihon University School of Dentistry at Matsudo, Chiba, Japan; 6 Iwate Dental Association, Iwate, Japan

**Keywords:** Mortality, Masticatory dysfunction, Teeth, Serum albumin, Older people

## Abstract

**Background:**

The association between dental status and mortality in community-dwelling older adults has been documented by several studies. The aim of this study was to analyze the contribution of self-assessed chewing ability, number of remaining teeth and serum albumin levels to mortality and the interactions between the three factors.

**Methods:**

A 20-year follow-up study was conducted with 666 subjects aged 80 years (from 1996 to 2017) who resided in the 8 areas served by one health center in Iwate Prefecture. Health check-ups including physical fitness measurements were conducted at a meeting place or gymnasium. Medical interview and blood sampling were conducted by physician. Oral examination was examined by dentist. The number of remaining teeth, serum albumin levels, and self-assessed chewing ability were used as predictors of mortality.

**Results:**

Among the 608 subjects (233 men and 375 women) included in this study, only 12 subjects (1.97%) survived after 20 years of follow-up. For men, dental status and serum levels of albumin were significantly associated with mortality. The hazard ratios of self-assessed chewing ability calculated by item response theory analysis and the inability to chew at least one food adjusted for serum albumin and tooth conditions were statistically significant in men. When adjusted by health status evaluated by blood tests, self-assessed chewing ability was statistically significant in men. According to path analysis, self-assessed chewing ability and serum albumin independently affected mortality in men.

**Conclusion:**

Masticatory dysfunction may be an important risk factor for mortality in men, even though it was self-assessed. Retaining chewing ability might be a useful predictor of longevity in older male adults.

## Background

The association between dental status and mortality in community-dwelling older adults has been documented by several studies [1–3]. Older adults with adequate dental status have a lower relative mortality risk than those with an inadequate dental status [4]. Two biological pathways have been proposed. One is related to odontogenic bacteremia, which causes chronic inflammatory damage in the whole body [5–11]. Another pathway is that an inadequate dental status affects nutritional status and finally leads to mortality. Tooth loss is directly associated with reduced masticatory efficiency, which leads to reduced and limited intake of food [12, 13]. In particular, a reduced consumption of fruits and vegetables [14–16], an increased intake of higher-fat and lower-fiber foods [17] and a lack of total protein and vitamins [18] are prominent in individuals with tooth loss [12, 13]. With an increased tendency towards consuming processed food rather than raw healthy food, the resulting carbohydrate-rich diet may increase mortality [19–21]. Nutritional status may be improved by the use of dentures [22]. Wearing dentures compensates for reduced mastication efficiency and improves mortality [23–25]. Serum levels of albumin, which represent nutritional status, was associated with mortality in older people [26]. These evidences support this pathway.

Although several studies have suggested an association between dental status and mortality, the observational periods have varied between the studies, and the studies did not completely evaluate the dental status, mastication efficiency, nutritional status and mortality of community-dwelling older adults. The evaluation of mortality and its risk factors in a uniform population is desirable. In this study, we analyzed the mortality of a uniform (80 years old) community-dwelling older adult population with a 20-year follow-up. We focused on the following risk factors for mortality: self-assessed chewing ability, number of teeth and serum albumin levels as nutritional status. Additionally, the interactions between these factors were analyzed.

## Methods

### Setting

A 20-year follow-up study was conducted with subjects aged 80 years old (from 1996 to 2017) residing in the 8 districts served by one health center in Iwate Prefecture.

### Study population

In 1997, the Japanese Ministry of Labor and Health directed and supported a survey of 80-year-old people residing in four areas in Japan. The aims of the survey were to investigate the relationship between oral health and systemic health in 80-year-old adults. This survey is known as the 8020 Data Bank Survey. Iwate Prefecture, located in the northern region of Japan, was one of the areas participating in this survey. The sampling method was cluster sampling, and the sampling frame was a complete count survey for all the subjects aged 80 years in 1997 (born in 1917) who resided in eight districts in Iwate Prefecture served by one public health center.

Based on the residential registration, public health nurses visited a total of 944 homes in which 80-year-olds lived. Public health nurses recommended that all 80-year-old individuals participate in the survey. Among the 80-year-old individuals, 814 agreed to participate. The surveys including oral examination, blood sampling, medical interview, and physical fitness test were conducted at a meeting place or gymnasium owned by the local government, and 666 subjects participated in these checkups. The 148 older adults who could not visit the checkup location were surveyed during a home visit by a health checkup team, which included a medical doctor and dentist. However, some individuals residing in nursing homes and hospitals refused to participate. Of the 666 subjects who attended a visit at a meeting place or gymnasium, 608 (233 men and 375 women) completed a questionnaire regarding lifestyle, oral health and systemic health, and they also underwent physical, laboratory, and oral examinations. No institutionalized older people were included in the analysis.

### Baseline data

The baseline survey was performed in 1996 at eight meeting halls or gymnasiums that were owned by the local government. The health checkups included an oral examination, medical examinations, blood tests, and physical fitness tests. Demographic and lifestyle factors were assessed during an interview based on the questionnaire.

### Follow-up study

In October 2017, the public health nurse surveyed the participants’ survival and date of death using the census register.

### Variables

The number of remaining teeth, serum level of albumin, body mass index (BMI), smoking status, alcohol consumption, and self-assessed chewing ability were used as the variables.

Medical interviews and blood collection were performed by a physician at a meeting place or gymnasium. Collected blood samples were kept at 4 °C and transferred to medical laboratory. Serum albumin and conventional health checkup items (Total protein, Aspartate Aminotransferase (AST), Alanine aminotransferase (ALT), γ-glutamyl transpeptidase (γ-GTP) Creatinine, Total cholesterol, Try glyceride, Blood glucose, Ig G, Ig A, Ig M, Calcium, Phosphate and Rheumatoid factor) were measured at medical laboratory. Number of remaining teeth, denture use and with or without dental caries were examined by dentist at a meeting place or gymnasium using dental mirror and probe under the light. Subjects were laid on temporary bed apparatus.

Smoking status, alcohol consumption, and self-assessed chewing ability were investigated by questionnaire. The questionnaire used in this study contained similar items as the 8020 Data Bank surveys conducted by the administration of the Ministry of Labor and Health in 1987 [18]. One week prior to the check-ups, the questionnaire was sent by mail. The questionnaire was collected before the medical check-ups. If missing data existed, the missing data were obtained by interviews based on the questionnaires. Physical fitness measurements were carried out only for subjects who were sufficiently fit, based on electrocardiograms or a physician’s interview [27].

BMI was calculated by standard method: weight (kg)/[height(m)]^2^. BMI was evaluated by the International Classification of adult underweight, overweight and obesity according to BMI (severe thinness: < 16, moderate thinness: 16 - < 17, mild thinness: 17 - < 18.5, normal range: 18.5 - < 25, obese class I: 25- < 30, obese class I: 30- < 35, obese class III: 35 - < 40) [28].

Smoking status was evaluated as current, previous or never, and alcohol consumption was evaluated as daily, more than 3 days per week, one or 2 days per week, less than 3 days per month, almost never, or never.

Self-assessed chewing ability was evaluated based on the following question regarding 15 different types of foods: Can you chew any of the following 15 types of food?’ The response was a simple dichotomous choice (yes/no). “No” indicate the chewing difficulty of the food. The validity of this self-reported chewing ability questionnaire has already been confirmed [29], and it has been applied in epidemiological studies [29, 30].

The conventional criteria were described previously [30]: 15 different types of food were divided into four groups, ranging from very-hard-to-chew to easy-to-chew foods. Three foods were very hard to chew (hard rice crackers, peanuts, and yellow pickled radish), six foods were moderately hard to chew (French bread, beefsteak, octopus in vinegar, pickled shallots, dried scallops, and dried cuttlefish), three foods were slightly hard to chew (konnyaku-jelly, a tubular roll of boiled fish paste, and squid sashimi), and three foods were easy to chew (boiled rice, tuna sashimi, and grilled eel).

Masticatory dysfunction is defined as a self-reported symptom or an objective deficit in the ability to chew a selected food [31]. In this study, we considered that subjects who were unable to chew at least one food among the 15 foods were had masticatory dysfunction.

### Statistical analysis

For the evaluation of the chewing difficulty of each food in the self-assessed chewing ability questionnaire, item response theory (IRT) was applied. A three-parameter logistic model was used. The item difficulty, item discrimination and asymptotes were calculated for each food and the scores of individual subjects, and an item response curve was constructed for each food.

Kaplan-Meier analysis was used to calculate the survival rate. To compare the significant differences in the survival curves, the log-rank test was used with or without risk factors assessed separately for men and women. Cox’s proportional hazard model was applied to calculate the hazard ratios of the number of remaining teeth, type of food and number of chewable foods categorized by self-assessed chewing difficulties. The hazard ratios of the number of remaining teeth, edentulous/dentulous status, serum albumin levels, BMI, smoking status, and alcohol intake, which were confounder candidates, were also calculated by Cox’s proportional hazard model. To determine the interrelationships of the factors significantly associated with mortality, path analysis was carried out. First, all paths were connected to mortality. Then, statistically insignificant paths were removed. To compare men and women, multiple-group structural equation modeling was carried out. The differences in statistical significance by sex were evaluated for each path in the final model.

R software with the irtoys package was used for the analysis of the item response theory. SPSS statistics Ver 24.0 (IBM, Tokyo, Japan) was used for the survival analysis, and AMOS Ver 24.0 was used for the path analysis.

## Results

Among the 608 subjects (233 men and 375 women) included in this study, only 12 subjects (1.97%) survived after 20 years of follow-up, and they had become centenarians. The baseline demographic characteristics of the subjects at the age of 80 are shown in Table 1. Descriptive statistics of self-assessed chewing ability are shown in Table S1.

**Table 1:** Demographic characteristics of the subjects participating in this stud

		Men (*n* = 233)	Women (*n* = 375)	*P*-value	Total
Continuous variable					
Number of remaining teeth	Mean SD	7.20 ± 8.71	3.09 ± 5.91	< 0.001	4.67 ± 8.70
	Median	3	0		0
	(25th–75th)	(0–13)	(0–3)		(0–8)
Serum levels of albumin (g/dL)	Mean SD	4.20 ± 0.31	4.20 ± 0.26	0.906	4.20 ± 0.31
	Median	4.2	4.2		4.2
	(25th–75th)	(4.00–4.40)	(4.10–4.40)		(4.10–4.40)
BMI	Mean SD	22.70 ± 3.26	23.67 ± 3.64	0.003	23.29 ± 3.26
	Median	22.66	23.47		23.11
	(25th–75th)	(20.28–24.73)	(21.36–25.92)		(20.83–25.57)
Life expectancy (days)	Mean SD	2641 ± 1483	2930 ± 1582	0.017	2819 ± 1550
	Median	2502	2711		2603
	(25th–75th)	(1707–3605)	(1830–3699)		(1798–3694)
Categorical variables					
Dentulous or edentulous					
Edentulous		104 (44.6%)	243 (64.8%)	< 0.001	347 (57.1%)
Dentulous		129 (55.4%)	132 (35.2%)		261 (42.9%)
Self-reported smoking status					
Current		57 (24.7%)	9 (2.4%)	< 0.001	66 (11.0%)
Previous or never		174 (75.3%)	361 (97.5%)		535 (89.0%)
Self-reported alcohol consumption					
Daily		78 (31.1%)	23 (5.6%)	< 0.001	101 (15.3%)
More than three days per week		9 (3.6%)	6 (1.5%)		15 (2.3%)
One or two days per week		29 (11.6%)	16 (3.9%)		45 (6.8%)
Less than 3 days per month		6 (2.4%)	9 (2.2%)		15 (2.3%)
Almost never		35 (13.9%)	35 (8.5%)		70 (10.6%)
Never		75 (29.9%)	283 (68.9%)		358 (54.1%)
Missing		19 (7.6%)	39 (9.5%)		58 (8.8%)
BMI					
Severe thinness		1 (0.5%)	0 (0.0%)	0.098	1 (0.2%)
Moderate thinness		3 (1.4%)	5 (1.5%)		8 (1.5%)
Mild thinness		13 (6.1%)	18 (5.4%)		31 (5.6%)
Normal range		148 (69.2%)	204 (60.7%)		352 (64.0%)
Preobese		46 (21.5%)	90 (26.8%)		136 (24.7%)
Obese class I		3 (1.4%)	17 (5.1%)		20 (3.6%)
Obese class II		0 (0.0%)	2 (0.6%)		2 (0.4%)

For continuous variables, differences by sex were evaluated by Mann-Whitney U tests, as the data were not normally distributed in the Kolmogorov-Smirnov results

For categorical variables, *p*-values were calculated by chi-square tests

Differences between men and women in life expectancy were statistically significant. Differences between men and women in the number of remaining teeth and “dentulous or edentulous” were statistically significant

We first analyzed mortality by demographic factors that may act as confounders for self-assessed chewing ability. Table 2 shows the hazard ratios of demographics and risk factors for mortality using Cox proportional hazard models. As it is well known that sex is strongly associated with mortality, hazard ratios were calculated separately by sex. For men, dental status and serum levels of albumin were statistically significant; in contrast, only serum levels of albumin were statistically significant in women. Underweight BMI levels in men were statistically significant when the normal range was used as a reference.

**Table 2:** Hazard ratios of demographics and risk factors estimated by Cox’s proportional hazard model for mortality

	Men			Women			Total			Strata model (Strata by sex)		
	Hazard Ratio (95% CI)	P-value	Model fit	Hazard Ratio (95% CI)	P-value	Model Fit	Hazard Ratio (95% CI)	*P*-value	Model fit	Hazard Ratio (95% CI)	*P*-value	Model fit
Number of remaining teeth	0.98 (0.96–0.99)	0.041	0.040	1.01 (0.99–1.03)	0.655	0.655	1.00 (0.99–1.02)	0.627	0.627	0.99 (0.97–1.00)	0.148	0.148
Edentulous/dentulous	1.52 (1.13–2.04)	0.006	0.006	1.12 (0.83–1.50)	0.461	0.461	1.09 (0.76–1.30)	0.437	0.437	1.30 (1.05–1.60)	0.016	0.016
Serum albumin (g/dL)	2.14 (1.42–3.24)	0.007	0.007	2.01 (1.15–3.52)	0.015	0.016	2.30 (1.30–4.24)	0.001	0.001	0.47 (0.31–0.71)	< 0.001	< 0.001
Smoking	0.86 (0.62–1.18)	0.180	0.180	1.42 (0.56–3.81)	0.492	0.490	0.79 (0.56–1.11)	0.140	0.140	0.66 (0.62–1.18)	0.341	0.341
BMI												
Normal range (18.5 ≤ BMI < 25)	Reference		0.007	Reference		0.777	Reference		0.402	Reference		0.292
Underweight (< 18.5)	1.90 (1.10–3.26)	0.021		0.81 (0.49–1.44)	0.478		1.07 (0.73–1.59)	0.720		1.12 (0.79–1.73)	0.445	
Obese class (25 ≤ BMI)	0.72 (0.49–1.05)	0.088		0.97 (0.71–1.34)	0.871		0.86 (0.68–1.10)	0.224		0.86 (0.68–1.10)	0.225	
Alcohol												
Never	Reference		0.853	Reference		0.278	Reference		0.189	Reference		0.511
Almost never	0.88 (0.56–1.38)	0.570		1.12 (0.70–1.81)	0.635		1.18 (0.86–1.62)	0.304		0.97 (0.70–1.35)	0.857	
Sometimes	0.88 (0.58–1.35)	0.566		0.63 (0.37–1.05)	0.077		0.93 (0.68–1.26)	0.623		0.79 (0.57–1.10)	0.137	
Daily	0.86 (0.61–1.23)	0.414		1.13 (0.64–1.99)	0.681		1.28 (0.98–1.66)	0.068		0.91 (0.68–1.20)	0.526	

For alcohol consumption, sometimes there were combinations of three categories: “More than three days per week”, “One or two days per week,” and “Less than 3 days per month”

For men, dental status and serum levels of albumin were statistically significant; in contrast, only serum levels of albumin were statistically significant in women

Almost all of the indexes were statistically significant in men except for smoking and alcohol intake. In contrast, only serum levels of albumin by clinical cut off were significant in women

Life expectancy has sex differences, and the hazard ratio of sex was a strong confounder in the investigation of the factors affecting mortality. This violates the hazard ratios of the factors being investigated. Using the stratified Cox proportional hazards model, hazard ratios of the factors investigated were assumed to be constant across the strata. The results of the strata model were adjusted for the hazard ratio of sex

For the strata model (strata by sex), edentulous/dentulous status and serum levels of albumin were statistically significant

Health status at the age of 80 was evaluated by the blood tests. Crude hazard ratios Ig G of men, Creatinine and Blood glucose of women were statistically significant. Therefore, chewing ability and serum albumin level were analyzed by multivariate adjusted Cox’s proportional hazard model. Chewing ability and serum levels albumin was statistically significant for the mortality after adjustment by blood tests. The results were shown in S2 Table.

Hazard ratios of masticatory dysfunction (inability to chew at least one food) are shown in Table 3. The number of chewable foods in all the groups was statistically significant in men.

**Table 3:** Hazard ratios of self-assessed chewing ability and self-assessed number of chewable foods categorized as hard, moderately hard, **slightly hard or easy**

	Men			Women			Total			Strata model (Strata by sex)		
	Hazard Ratio (95% CI)	P-value	Model fit	Hazard Ratio (95% CI)	*P*-value	Model fit	Hazard Ratio (95% CI)	P-value	Model fit	Hazard Ratio (95% CI)	P-value	Model fit
Self-assessed chewing ability by item response theory												
1.17 (1.07–1.27)		< 0.001	< 0.001	1.01 (0.92–1.09)	0.937	0.791	1.03 (0.98–1.08)	0.298	0.298	1.07 (1.02–1.14)	0.013	0.013
Self-assessed inability to chew at least one food among the 15 different types of food												
1.72 (1.20–2.46)		0.028	0.003	0.85(0.59–1.21)	0.361	0.360	1.10 (0.83–1.35)	0.652	0.625	1.25(0.97–1.62)	0.088	0.088
Self-assessed number of very hard-to-chew foods												
3	Reference		0.001	Reference		0.307	Reference		0.011	Reference		0.002
2	1.04 (0.72–1.50)	0.830		1.17 (0.82–1.66)	0.401		1.10 (0.86–1.43)	0.445		1.10(0.85–1.42)	0.460	
1	0.89 (0.45–1.75)	0.730		0.93 (0.59–1.46)	0.745		0.82 (0.57–1.20)	0.308		0.93(0.64–1.35)	0.686	
0	2.43 (1.56–3.79)	< 0.001		1.40 (0.95–2.06)	0.093		1.55 (1.16–2.07)	0.003		1.72(1.28–2.31)	< 0.001	
Self-assessed number of moderately hard-to-chew foods												
6	Reference		0.002	Reference		0.444	Reference		0.645	Reference		0.240
5	1.50 (0.92–2.44)	0.102		0.66 (0.39–1.10)	0.101		0.90 (0.64–1.28)	0.548		1.02(0.71–1.45)	0.921	
4	1.42 (0.85–2.39)	0.184		1.00 (0.62–1.61)	0.994		1.10 (0.77–1.55)	0.594		1.23(0.87–1.76)	0.950	
3	1.34 (0.84–2.12)	0.217		0.72 (0.45–1.16)	0.177		0.89 (0.64–1.23)	0.464		1.01(0.73–1.41)	0.950	
2	1.39 (0.87–2.22)	0.166		1.09 (0.70–1.70)	0.706		1.15 (0.84–1.59)	0.375		1.27(0.92–1.76)	0.140	
1	1.95 (1.10–3.47)	0.023		0.96 (0.60–1.51)	0.845		1.07 (0.76–1.52)	0.697		1.34(0.93–1.92)	0.115	
0	3.82 (2.03–7.18)	< 0.001		0.90 (0.52–1.54)	0.690		1.21 (0.81–1.80)	0.365		1.55(1.02–2.36)	0.038	
Self-assessed number of slightly hard-to-chew foods												
3	Reference		< 0.001	Reference		0.532	Reference		0.098	Reference		0.006
2	1.76(1.16–2.65)	0.007		0.97(0.69–1.36)	0.853		1.06(0.82–1.38)	0.645		1.22(0.93–1.59)	0.149	
1–0	6.31(3.09–12.87)	< 0.001		1.27(0.76–2.14)	0.360		1.53(1.01–2.30)	0.042		1.89(1.24–2.88)	0.003	
Self-assessed number of easy-to-chew foods												
3	Reference		0.003	Reference		0.116	Reference		0.194	Reference		0.798
0–2	1.72(1.05–2.62)	0.030		0.752(0.53–1.07)	0.117		0.83(0.63–1.10)	0.194		0.96(0.72–1.28)	0.798	

The fifteen foods investigated in this study were classified as hard, moderately hard, slightly hard and easy. Several statistically significant hazard ratios were observed. However, only slightly hard food had a dose-response relationship. When combining the results for 0 and 1, the hazard ratio was 1.757 (1.164–2.652) (*p* = 0.007). Model fit indicates the p-values calculated by the Wald test

**Table 4:** Multivariate-adjusted hazard ratios for mortality

	Men			Women			Total			Strata model (Strata by sex)		
	Hazard Ratio (95% CI)	*P*-value	Model fit	Hazard Ratio (95% CI)	P-value	Model fit	Hazard Ratio (95% CI)	P-value	Model fit	Hazard Ratio (95% CI)	*P*-value	Model fit
Serum albumin (mg/dL)	0.44 (0.24–0.81)	0.008	< 0.001	0.51 (0.29–0.89)	0.019	0.110	0.49 (0.32–0.73)	0.001	0.006	0.46 (0.31–0.69)	< 0.001	0.001
Chewing ability (IRT)	0.89 (0.81–0.96)	0.006		1.01 (0.94–1.10)	0.746		0.98 (0.92–1.03)	0.439		0.95 (0.90–1.01)	0.077	
Number of remaining teeth	0.99 (0.97–1.01)	0.277		1.00 (0.98–1.03)	0.816		1.00 (0.99–1.02)	0.557		0.99 (0.98–1.01)	0.342	
Serum albumin (mg/dL)	0.46 (0.25–0.83)	0.011	< 0.001	0.48 (0.27–0.85)	0.012	0.082	0.48 (0.32–0.72)	<.0001	0.005	0.45 (0.30–0.68)	< 0.001	< 0.001
Chewing ability (IRT)	0.89 (0.81–0.97)	0.006		1.01 (0.94–1.10)	0.744		0.98 (0.93–1.04)	0.556		0.95 (0.89–1.00)	0.070	
Edentulous/dentulous	1.37 (0.53–1.89)	0.054		1.15 (0.83–1.59)	0.396		1.08 (0.66–1.33)	0.493		1.29 (1.02–1.59)	0.032	
Serum albumin (mg/dL)	0.45 (0.25–0.82)	0.009	0.001	0.51 (0.29–0.90)	0.021	0.099	0.49 (0.32–0.73)	0.001	0.006	0.46 (0.31–0.70)	< 0.001	0.001
Chewing ability (inability at least one food)	0.63 (0.43–0.91)	0.014		1.12 (0.77–1.62)	0.547		0.92 (0.71–1.20)	0.548		0.81 (0.62–1.06)	0.131	
Number of remaining teeth	0.99 (0.97–1.01)	0.226		1.00 (0.98–1.03)	0.821		1.00 (0.99–1.02)	0.592		0.99 (0.98–1.01)	0.307	
Serum albumin (mg/dL)	0.47 (0.26–0.85)	0.012	< 0.001	0.48 (0.27–0.86)	0.013	0.074	0.48 (0.32–0.72)	< 0.001	0.006	0.45 (0.30–0.68)	< 0.001	< 0.001
Chewing ability (inability at least one food)	0.63 (0.43–0.91)	0.013		1.12 (0.77–1.62)	0.561		0.94 (0.73–1.22)	0.658		0.81 (0.62–1.06)	0.118	
Edentulous/dentulous	0.72 (0.52–0.99)	0.041		0.87 (0.64–1.20)	0.405		0.93 (0.75–1.15)	0.478		0.78 (0.62–0.98)	0.029	
Serum albumin (mg/dL)	0.45 (0.24–0.83)	0.011	< 0.001	0.51 (0.29–0.89)	0.018	0.193	0.49 (0.32–0.73)	0.001	0.014	0.46 (0.30–0.69)	< 0.001	0.002
Slightly hard-to-chew foods (2)	1.68 (1.07–2.65)	0.025		0.94 (0.65–1.36)	0.740		1.04 (0.78–1.38)	0.778		1.17 (0.88–1.56)	0.285	
Slightly hard-to-chew foods (0–1)	5.59 (2.18–14.36)	< 0.001		1.05 (0.56–1.94)	0.887		1.22 (0.74–2.02)	0.442		1.46 (0.87–2.44)	0.150	
Number of remaining teeth	0.98 (0.96–1.00)	0.088		1 .00(0.98–1.03)	0.787		1.00 (0.99–1.02)	0.627		0.99 (0.98–1.01)	0.236	
Serum albumin (mg/dL)	0.47 (0.25–0.87)	0.016	< 0.001	0.47 (0.27–0.84)	0.011	0.149	0.48 (0.32–0.72)	< 0.001	0.012	0.45 (0.29–0.68)	< 0.001	< 0.001
Slightly hard-to-chew foods (2)	1.69 (1.07–2.66)	0.024		0.94 (0.65–1.37)	0.757		1.03 (0.78–1.37)	0.824		1.18 (0.88–1.57)	0.270	
Slightly hard-to-chew foods (0–1)	5.49 (2.14–14.07)	< 0.001		1.05 (0.57–1.95)	0.870		1.21 (0.73–2.00)	0.465		1.49 (0.89–2.49)	0.132	
Edentulous/dentulous	1.47 (1.06–2.00)	0.019		1.15 (0.83–1.59)	0.392		1.09 (0.87–1.33)	0.460		1.30 (1.04–1.61)	0.023	

Chewing ability (IRT): Self-assessed chewing ability calculated by the scores of the 3-parameter logistic model of item response theory

Chewing ability (inability to chew at least one food): Self-assessed inability to chew at least one food among the 15 different types of food

Self-assessed number of slightly hard foods that cannot be chewed among 3 foods

Serum albumin levels were statistically significant for all of the models. Masticatory function evaluation based on IRT analysis. Inability to chew at least one food among the 15 different types of food and self-assessed number of slightly hard foods that cannot be chewed among 3 foods were all statistically significant in men. However, they were not significant in women or the total population. They were not statistically significant in the strata model (strata by sex)

Mortality according to self-assessed ability to chew 15 types of different foods is shown in Table S3. The slightly hard food-to-chew food group consisted of konnyaku-jelly, a tubular roll of boiled fish paste, and squid-sashimi. Among the 15 foods, 10 foods had statistically significant hazard ratios for mortality in men. The hazard ratios of the three types of slightly hard-to-chew foods were all statistically significant. In contrast, no food had a statistically significant hazard ratio for women. To determine the characteristics of the three slightly hard-to-chew foods, an item response analysis was carried out. The results of the item response analysis and the item response curves are shown in Table S4 and Fig. S1. These foods had relatively high item discrimination and low item difficulty. In addition, according to the item response curves shown in Fig. S1, the conventional classification of foods by their hardness-to-chew characteristic was not completely consistent with the item difficulty.

Next, we evaluated the hazard ratios for men with multiple regression using Cox hazard models (Table 4). Masticatory dysfunction, serum albumin levels and tooth condition were used as the dependent variables.

As shown in Table 3, serum albumin levels were statistically significant in all of the models. Self-assessed chewing ability was statistically significant in men; however, it was not statistically significant in all of the models in women. Additionally, the survival curves of these four items are shown in Fig. S2. In men, the survival curves of individuals with these three risk factors were consistently located beneath those without risk factors. For edentulous subjects, wearing dentures was found to be important to maintain chewing ability. For men, not wearing dentures was a significant risk factor for mortality (Table S5). These results indicated that self-assessed chewing ability and serum levels albumin were important determining factors for the mortality of older men.

**Fig. 1:**
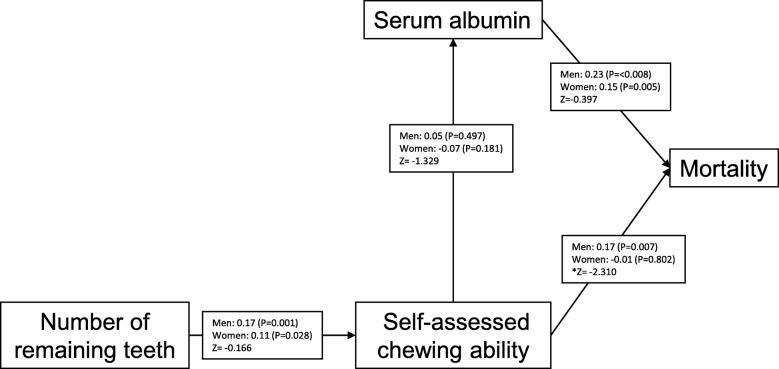
Path diagram of factors affecting mortality. The path coefficients are shown separately for men and women. The paths from self-assessed chewing ability to serum albumin were not statistically significant in men or women. Absolute Z values greater than 1.96 indicate that the paths are significantly different in men and women. In this case, the path from self-assessed chewing ability to mortality was significantly different in men and women. *: Statistically significant difference between men and women

Finally, to analyze the interrelationships and contribution to mortality of statistically significant risk factors using the results of Cox’s proportional hazard model (self-assessed chewing ability by IRT, dental status and serum levels of albumin), path analysis was carried out independently by sex. First, all paths were connected among serum albumin, number of remaining teeth, self-assessed chewing ability and mortality. Then, statistically insignificant paths were removed. The final model is presented in Fig. 1. The path from self-assessed chewing ability to mortality was statistically significant in men but not in women, and it was significantly different by sex. The path from serum albumin to mortality was statistically significant in both men and women. The path from the number of remaining teeth to self-assessed chewing ability was statistically significant in both men and women. The path from self-assessed chewing ability to serum albumin was not significant in either men or women. The results indicated that self-assessed chewing ability and serum albumin independently affected mortality.

## Discussion

In this study, we found that self-assessed chewing ability may be the most important predictor of mortality in men. Self-assessed chewing ability was statistically significant even after adjusting for serum albumin and the number of remaining teeth. Based on the path analysis, serum levels of albumin and self-assessed chewing ability acted independently on mortality.

Masticatory dysfunction is directly related to malnutrition. Subjects with fewer remaining teeth and inadequate prostheses had worse nutritional status with regards to dietary quality [32] and intake of nutrients [33]. It is well known that the serum level of albumin is an important predictor of mortality among older adults [34–38]. To the best of our knowledge, there is only one report that simultaneously investigated serum levels of albumin and masticatory dysfunction [30]. The results of that study showed that masticatory dysfunction acted independently of serum levels of albumin on the risk of mortality. Nutrition is not only a matter of chewing ability but also depends on other influencing factors [39], and a systematic review concluded that chewing ability explains only part of the variance in food and nutrient intake by older adults [40].

Several studies focused on the number of remaining teeth or periodontal status and cardiovascular mortality [41, 42]. The pathway is related to odontogenic bacteremia. Number of remaining teeth of the subjects participated in this study was very small. Edentulous subject was periodontally healthy by the conventional methodology of evaluation, though they lost their teeth by periodontal disease. To inspect this pathway, cohort study that follow up from younger ages is necessary. In this study, number of remaining teeth was not statistically significant for the mortality. Japanese national insurance system covers the prosthodontic treatment including full dentures. Many edentulous subjects participated in this study wear dentures. The number of functional teeth calculated according to the previous report, 78% (474/606%) of the subjects had 28 functional teeth. Therefore, the effect of number of remaining teeth that leaded to chewing ability may be masked by the prothesis.

This study focused on the healthy older people who can attend the health checkups held at a meeting place or gymnasium. Most of the general health status evaluated blood test had no statistically significant effect on the mortality. When focused on the specific cause of death and blood test, significant effect may be obtained. Only serum levels albumin and creatinine were statistically significant for both men and women. Serum levels albumin reflect nutritional status and creatinine reflect glomerular filtration of kidney. As discussed above, nutritional status close connection with oral function. Kidney had three major function: blood pressure control, production red blood cells, and calcium control. These functions are not easy to directly connect with oral function. Further profound study is to prove these interactions. However, multivariate adjusted chewing ability had statistically significant for the mortality. Kidney functions and oral functions may independently effect on the mortality.

One of the limitations of this study was that masticatory dysfunction was investigated by self-assessment. In epidemiological studies, self-assessed masticatory dysfunction by a specific questionnaire has been commonly used because of its simple and easy handling and cost effectiveness. Subjective methods require specific devices and a greater work force and cost than the questionnaire method. In this study, similar hazard ratios were obtained using the simple questions: “Do you experience difficulty chewing?”, “Has the amount of food you usually eat decreased in the last year because of chewing problems?” [39] and “Can you chew any type of food?” [43, 44]. In epidemiological surveys, self-assessed mastication can meet the demands for the prediction of mortality.

The number of remaining teeth was significant for mortality only in men. Edentulous subjects were at significant risk when dentulous subjects were used as a reference. Several studies have shown that the number of remaining teeth had a statistically significant association with mortality in older adults. However, some of the studies failed to show clear dose-response relations [43, 44]. This may be because the effects of tooth loss can be compensated for with adequate dentures. Therefore, tooth loss was classified in combination with denture use, and significant improvements resulted [25, 45]. Hazard ratios for the edentulous subjects or the subjects with fewer than 20 teeth were statistically significant when subjects with more than 20 teeth were used as the reference [46–49]. In this study, the number of remaining teeth did not directly influence mortality. Therefore, the number of remaining teeth should be considered one of the indicators of oral function.

There is a sex difference in mortality related to the number of remaining teeth [48, 50–53]. Most studies have shown that tooth loss is a risk factor for mortality in males and not in females [47, 51, 52]. Our results are consistent with these studies. Other studies have shown contradictory results [50, 53]. Follow-up periods, the baseline number of remaining teeth, and statistical methods were different between studies. In addition, mortality is a multifactorial issue, and some related factors cause either tooth loss or mortality. In particular, socioeconomic status may be an important factor for mortality. In this study, we could not obtain socioeconomic status data. Therefore, it is impossible to reach a clear conclusion on the effect of the interaction of the number of remaining teeth with sex on mortality.

Another important confounding factor concerning the number of remaining teeth is the use of dentures. Wearing dentures compensates for deteriorated oral functioning. Wearing dentures improved mortality [24, 25, 39]. A recently published systematic review noted that there was no report that compared mortality in complete denture wearers to mortality in edentulous patients who were not wearing complete dentures [54]. As shown in Table S4, not wearing complete dentures was a risk factor for mortality in edentulous men.

There are several limitations in this study. There were no data on socioeconomic status or education levels. In Japan, the national pension system supplies living expenses for people over 65 years old. The system covers the whole nation. The Public Assistance Act protects the livelihood of low-income people. In addition, national medical insurance covers the whole nation. However, it does not include information on socioeconomic status in this population. A previous study suggested that the number of teeth was a significant predictor of mortality independent of health factors, socioeconomic status and lifestyle [53]. The effect of socioeconomic status or education levels should be confirmed. Another limitation was the combinations of the categories of self-assessed chewing ability. Cells with fewer than 10 counts were combined for the Cox proportional hazard analysis.

In summary, masticatory dysfunction may be an important risk factor for mortality in males, even though it was self-assessed. In particular, slightly hard-to-chew food can be an indicator of masticatory dysfunction to predict mortality in older males. In addition, the number of remaining teeth was an indicator of chewing ability. For edentulous older males, not using dentures can be a risk factor for mortality. Although chewing ability was self-assessed, this method is very convenient for use in public health studies. In particular, it may be applicable for the screening of masticatory dysfunctions in men.

## Conclusion

Retaining chewing ability might be a useful indicator of longevity in older male adults.

## Supplementary information

**Additional file 1: Table S1.** Frequency of subjects who cannot chew each food (A) Frequency of subjects who cannot chew each food.

**Additional file 2: Table S2.** Hazard ratios of health status and chewing ability.

**Additional file 3: Table S3.** Hazard ratios of the self-assessed ability to chew 15 foods.

**Additional file 4: Table S4.** Item parameter estimates for the 3-parameter logistic model.

**Additional file 5: Table S5.** Effect of the use of dentures on the mortality of edentulous subjects adjusted by serum albumin levels.

**Additional file 6: Figure S1.** Item response curves for the 15 different types of foods. **Figure S2** Survival curves for serum albumin, edentulous/dentulous status and masticatory dysfunction.

## Data Availability

All relevant data are strictly administered by the 8020 Foundation associated with the Japan Dental Association and Ministry of Health and Welfare of Japan. To use the data from the “8020 Data Bank”, application to and approval by the administrative board is necessary.

## Abbreviations

AST: Aspartate Aminotransferase
ALT: Alanine aminotransferase
γ-GTP: γ-glutamyl transpeptidase
BMI: Body mass index
IRT: Item response theory
