# Supplementary material for: Effects of self-assessed chewing ability, tooth loss and serum albumin on mortality in 80-year-old individuals: a 20-year follow-up study
Source: BMC Oral Health. 2020 Apr 21;20:122. doi: 10.1186/s12903-020-01113-7 (PMC7175538; doi:10.1186/s12903-020-01113-7)
Supplement: Supplementary file 1 — Additional file 1: Table S1. Frequency of subjects who cannot chew each food (A) Frequency of subjects who cannot chew each food. [file 12903_2020_1113_MOESM1_ESM.docx]

**Table S1 Frequency of subjects who cannot chew each food**

**(A) Frequency of subjects who cannot chew each food**

|  | Men (n=233) | Women (n=375) | P-value | Total |
| --- | --- | --- | --- | --- |
|  | n (%) | n (%) |  | n (%) |
| Very hard-to-chew food | | | | |
| Peanuts | 46 (19.7%) | 107 (28.5%) | 0.015 | 153 (25.2%) |
| Yellow pickled radish | 55 (23.6%) | 116 (30.9%) | 0.051 | 171 (28.1%) |
| Hard rice crackers | 59 (25.3%) | 104 (27.7%) | 0.514 | 163 (26.8%) |
| Moderately hard-to-chew food | | | | |
| French bread | 91 (39.1%) | 177 (47.2%) | 0.049 | 268 (44.1%) |
| Beefsteak | 69 (29.6%) | 167 (44.5%) | <0.001 | 236 (38.8%) |
| Octopus in vinegar | 86 (36.9%) | 173 (46.1%) | 0.025 | 259 (42.6%) |
| Pickled shallots | 40 (17.2%) | 84 (22.4%) | 0.120 | 124 (20.4%) |
| Dried scallops | 116 (49.8%) | 221 (58.9%) | 0.027 | 337 (55.4%) |
| Dried cuttlefish | 115 (49.4%) | 228 (60.8%) | 0.006 | 343 (56.4%) |
| Slightly hard-to-chew food | | | | |
| Konnyaku-jelly | 12 (5.2%) | 22 (5.9%) | 0.709 | 34 (5.6%) |
| Tubular roll of boiled fish paste | 13 (5.6%) | 22 (5.9%) | 0.882 | 35 (5.8%) |
| Squid-sashimi | 32 (13.7%) | 101 (26.9%) | <0.001 | 133 (21.9%) |
| Easy-to-chew food | | | | |
| Steamed rice | 8 (3.4%) | 0 (0%) | <0.001 | 8 (1.3%) |
| Tuna sashimi | 5 (2.1%) | 27 (7.2%) | 0.007 | 32 (5.3%) |
| Grilled eel | 15 (6.4%) | 57 (15.2%) | 0.001 | 72 (11.8%) |

For the differences between sexes, p-values were calculated by chi-square tests.

**(B) Frequency of subjects by number of foods categorized as very hard, moderately hard, slightly hard, and easy to chew**

|  | Men (n=233) | Women (n=375) | P-value | Total |
| --- | --- | --- | --- | --- |
| Very hard-to-chew food | | | | |
| 0 | 28 (12.0%) | 55 (14.7%) | 0.077 | 83 (13.7%) |
| 1 | 14 (6.0%) | 43 (11.5%) |  | 57 (9.4%) |
| 2 | 48 (20.6%) | 76 (20.3%) |  | 124 (20.4%) |
| 3 | 143 (61.4%) | 201 (53.6%) |  | 344 (56.6%) |
| Moderately hard-to-chew food | | | | |
| 0 | 15 (6.4%) | 42 (11.2%) | 0.026 | 57 (9.4%) |
| 1 | 19 (8.2%) | 56 (14.9%) |  | 75 (12.3%) |
| 2 | 33 (14.2%) | 51 (13.6%) |  | 84 (13.8%) |
| 3 | 37 (15.9%) | 60 (16.0%) |  | 97 (16.0%) |
| 4 | 29 (12.4%) | 44 (11.7%) |  | 73 (12.0%) |
| 5 | 31 (13.3%) | 46 (12.3%) |  | 77 (12.7%) |
| 6 | 69 (29.6%) | 76 (20.3%) |  | 145 (23.8%) |
| Slightly hard-to-chew food | | | | |
| 0 | 4 (1.7%) | 13 (3.5%) | 0.051 | 17 (2.8%) |
| 1 | 6 (2.6%) | 13 (3.5%) |  | 19 (3.1%) |
| 2 | 33 (14.2%) | 80 (21.3%) |  | 113 (18.6%) |
| 3 | 190 (81.5%) | 269 (71.7%) |  | 459 (75.5%) |
| Easy-to-chew food | | | | |
| 1 | 3 (1.3%) | 15 (4.0%) | 0.005 | 18 (3.0%) |
| 2 | 22 (9.4%) | 54 (14.4%) |  | 76 (12.5%) |
| 3 | 208 (89.3%) | 306 (81.6%) |  | 514 (84.5%) |

The numbers indicate the sum of the count of self-assessed chewable food in each category.

P-values were calculated by chi-square tests for “Very hard-to-chew food”, “Moderately hard-to-chew food” and “Slightly hard-to-chew food”. For “Easy-to-chew food”, Fisher’s exact test was applied.
