# Supplementary material for: Effects of self-assessed chewing ability, tooth loss and serum albumin on mortality in 80-year-old individuals: a 20-year follow-up study
Source: BMC Oral Health. 2020 Apr 21;20:122. doi: 10.1186/s12903-020-01113-7 (PMC7175538; doi:10.1186/s12903-020-01113-7)
Supplement: Supplementary file 2 — Additional file 2: Table S2. Hazard ratios of health status and chewing ability. [file 12903_2020_1113_MOESM2_ESM.docx]

S2 Table Hazard ratios of health status and chewing ability

(A) Crude hazard ratios of the items of blood test

(B) Multivariate adjusted hazard ratios of chewing ability and items of blood test.

(A)

|  | Men | | | Women | | |
| --- | --- | --- | --- | --- | --- | --- |
|  | ^Hazard Ratio (95% CI)^ | ^P-value^ | ^Model fit^ | ^Hazard Ratio (95% CI)^ | ^P-value^ | ^Model fit^ |
| Total protein(g/dL) | 0.86 (0.61-1.23) | 0.94 | 0.42 | 1.01 (0.73-1.41) | 0.94 | <0.01 |
| AST ( U ) | 1.00 (1.00-1.01) | 0.42 | 0.24 | 1.00 (0.99-1.01) | 0.71 | 0.52 |
| ALT ( U ) | 1.00 (0.99-1.01) | 0.70 | 0.63 | 1.00 (0.99-1.01) | 0.66 | 0.98 |
| γ-GTP ( U ) | 1.00 (1.00-1.01) | 0.28 | 0.08 | 1.01 (0.99-1.02) | 0.24 | 0.86 |
| Creatinine (mg/dL) | 1.58 (0.91-2.76) | 0.65 | 0.10 | 1.63 (1.32-2.02) | <0.01 | 0.09 |
| Total cholesterol (mg/dL) | 1.00 (0.99-1.00) | 0.64 | 0.93 | 1.00 (1.00-1.01) | 0.26 | 0.50 |
| Try glyceride (mg/dL) | 1.00 (1.00-1.00) | 0.26 | 0.54 | 1.00 (1.00-1.00) | 0.92 | 0.94 |
| Blood glucose (mg/dL) | 1.00 (1.00-1.00) | 0.11 | 0.51 | 1.01 (1.00-1.01) | <0.01 | 0.93 |
| Ig G (mg/dL) | 1.00 (1.00-1.00) | <0.01 | 0.86 | 1.00 (1.00-1.00) | 0.98 | 0.38 |
| IgA (mg/dL) | 1.00 (1.00-1.00) | 0.13 | 0.50 | 1.00 (1.00-1.00) | 0.07 | 0.50 |
| IgM (mg/dL) | 1.00 (1.00-1.00) | 0.26 | 0.93 | 1.00 (1.00-1.00) | 0.94 | 0.71 |
| Calcium (mg/dL) | 1.32 (0.59-2.91) | 0.93 | 0.50 | 1.41 (0.66-3.02) | 0.38 | 0.07 |
| Phosphate (mg/dL) | 1.40 (0.97-2.04) | 0.92 | 0.07 | 1.07 (0.76-1.49) | 0.71 | 0.38 |
| Rheumatoid factor (IU/mL) | 1.00 (1.00-1.00) | 0.53 | 0.05 | 1.00 (1.00-1.01) | 0.34 | 0.11 |
| Maximal blood pressure (mm Hg) | 0.99 (0.97-1.00) | 0.33 | 0.34 | 1.00 (1.00-1.01) | 0.33 | 0.34 |
| Minimal blood pressure (mm Hg) | 1.00 (0.98-1.01) | 0.51 | 0.51 | 1.00 (0.98-1.01) | 0.69 | 0.69 |

(B)

|  | Men | | | Women | | |
| --- | --- | --- | --- | --- | --- | --- |
|  | ^Hazard Ratio (95% CI)^ | ^P-value^ | ^Model fit^ | ^Hazard Ratio (95% CI)^ | ^P-value^ | ^Model fit^ |
| Chewing Ability | 1.14 (1.04-1.25) | <0.01 | 0.05 | 1.04 (0.96-1.05) | 0.39 | 0.01 |
| Serum albumin (g/dL) | 7.14 (2.00-25.00) | <0.01 |  | 3.33 (1.52-7.14) | <0.01 |  |
| Total protein (g/dL) | 2.16 (0.75-6.26) | 0.16 |  | 1.35 (0.60-3.00) | 0.47 |  |
| AST ( U ) | 1.01 (1.00-1.03) | 0.15 |  | 1.01 (0.99-1.04) | 0.35 |  |
| ALT ( U ) | 0.99 (0.96-1.01) | 0.21 |  | 0.98 (0.95-1.01) | 0.19 |  |
| γGTP ( U ) | 1.01 (1.00-1.02) | 0.07 |  | 1.01 (0.99-1.03) | 0.39 |  |
| Creatinine (mg/dL) | 1.96 (1.09-3.54) | 0.03 |  | 1.61 (1.25-2.08) | <0.01 |  |
| Total cholesterol (mg/dL) | 1.00 (0.99-1.00) | 0.61 |  | 1.00 (1.00-1.01) | 0.21 |  |
| Try glyceride (mg/dL) | 1.00 (1.00-1.00) | 0.17 |  | 1.00 (1.00-1.00) | 0.57 |  |
| Blood glucose (mg/dL) | 1.00 (1.00-1.01) | 0.09 |  | 1.01 (1.00-1.01) | <0.01 |  |
| Ig G (mg/dL) | 1.00 (1.00-1.00) | 0.05 |  | 1.00 (1.00-1.00) | 0.31 |  |
| IgA (mg/dL) | 1.00 (1.00-1.00) | 0.35 |  | 1.00 (1.00-1.00) | 0.45 |  |
| IgM (mg/dL) | 1.00 (1.00-1.00) | 0.72 |  | 1.00 (1.00-1.00) | 0.89 |  |
| Calcium (mg/dL) | 2.79 (0.88-8.82) | 0.08 |  | 1.84 (0.63-5.35) | 0.26 |  |
| Phosphate (mg/dL) | 1.38 (0.90-2.11) | 0.14 |  | 1.14 (0.79-1.65) | 0.49 |  |
| Rheumatoid factor (IU/mL) | 1.00 (1.00-1.00) | 0.30 |  | 1.00 (0.99-1.01) | 0.71 |  |
| Maximal blood pressure (mm Hg) | 1.00 (0.99-1.01) | 0.73 |  | 1.01 (1.00-1.02) | 0.33 |  |
| Minimal blood pressure (mm Hg) | 1.01 (0.99-1.03) | 0.52 |  | 1.00 (0.98-1.01) | 0.60 |  |

Chewing ability was the ability by item response theory
