# Supplementary material for: Effects of self-assessed chewing ability, tooth loss and serum albumin on mortality in 80-year-old individuals: a 20-year follow-up study
Source: BMC Oral Health. 2020 Apr 21;20:122. doi: 10.1186/s12903-020-01113-7 (PMC7175538; doi:10.1186/s12903-020-01113-7)
Supplement: Supplementary file 3 — Additional file 3: Table S3. Hazard ratios of the self-assessed ability to chew 15 foods. [file 12903_2020_1113_MOESM3_ESM.docx]

**Table S3 Hazard ratios of the self-assessed ability to chew 15 foods**

|  | Men (n=233) | | | Women (n=375) | | | Total | | | Strata model (Strata by sex) | | |
| --- | --- | --- | --- | --- | --- | --- | --- | --- | --- | --- | --- | --- |
|  | Hazard Ratio  (95% CI) | P-value | Model Fit | Hazard Ratio (95% CI) | P-value | Model Fit | Hazard Ratio  (95% CI) | P-value | Model Fit | Hazard Ratio (95% CI) | P-value | Model Fit |
| Very hard-to-chew food | | | | | | | | | | | | |
| Peanuts | 1.44 (1.00 - 2.07) | 0.048 | 0.048 | 1.18 (0.88 - 1.59) | 0.273 | 0.273 | 1.28 (1.61 -2.03) | 0.038 | 0.038 | 1.28 (1.01 - 1.61) | 0.038 | 0.038 |
| Yellow pickled radish | 1.40 (0.99 - 1.96) | 0.055 | 0.055 | 1.18 (0.88 - 1.58) | 0.273 | 0.273 | 1.27 (1.01 - 1.58) | 0.039 | 0.039 | 1.27 (1.01 - 1.58) | 0.038 | 0.039 |
| Hard rice crackers | 1.60 (1.15 - 2.22) | 0.005 | 0.005 | 1.14 (0.85 - 1.54) | 0.389 | 0.389 | 1.32 (1.06 - 1.65) | 0.013 | 0.013 | 1.32 (1.06 - 1.65) | 0.013 | 0.013 |
| Moderately hard-to-chew food | | | | | | | | | | | | |
| French bread | 1.27 (0.95 - 1.71) | 0.108 | 0.108 | 1.14 (0.87 - 1.50) | 0.340 | 0.340 | 1.20 (0.98 - 1.47) | 0.074 | 0.074 | 1.21 (0.98 - 1.49) | 0.076 | 0.076 |
| Beefsteak | 1.44 (1.05 - 1.98) | 0.024 | 0.024 | 1.02 (0.81 - 1.40) | 0.644 | 0.644 | 1.21 (0.98 - 1.49) | 0.076 | 0.076 | 1.14 (0.93 - 1.40) | 0.196 | 0.196 |
| Octopus in vinegar | 1.25 (0.93 - 1.70) | 0.140 | 0.140 | 1.06 (0.81 - 1.39) | 0.671 | 0.671 | 1.14 (0.93 - 1.40) | 0.196 | 0.196 | 1.14 (0.88 - 1.48) | 0.324 | 0.324 |
| Pickled shallots | 1.83 (1.26 - 2.67) | 0.002 | 0.002 | 0.89 (0.57 - 1.16) | 0.258 | 0.258 | 1.14 (0.88 - 1.48) | 0.324 | 0.324 | 1.23 (1.00 - 0.50) | 0.049 | 0.050 |
| Dried scallops | 1.45 (1.08 - 1.94) | 0.014 | 0.014 | 1.06 (0.80 - 1.39) | 0.699 | 0.699 | 1.27 (1.00 - 1.50) | 0.050 | 0.050 | 1.17 (0.95 - 1.43) | 0.138 | 0.139 |
| Dried cuttlefish | 1.26 (1.02 - 1.85) | 0.036 | 0.036 | 1.01 (0.76 - 1.33) | 0.947 | 0.947 | 1.17 (0.95 - 1.43) | 0.139 | 0.139 | 1.96 (1.30 - 2.97) | 0.001 | 0.001 |
| Slightly hard-to-chew food | | | | | | | | | | | | |
| Konnyaku-jelly | 5.12 (2.68 - 9.80) | <0.001 | <0.001 | 1.30 (0.76 - 2.25) | 0.340 | 0.340 | 1.96 (1.30 - 2.97) | 0.001 | 0.001 | 1.20 (0.93 - 1.55) | 0.161 | 0.161 |
| Tubular roll of boiled fish paste | 2.80 (1.55 - 5.08) | 0.001 | 0.001 | 1.23 (0.71 - 2.12) | 0.454 | 0.454 | 1.67 (1.12 - 2.50) | 0.012 | 0.012 | 4.92 (2.36 - 10.28) | <0.001 | <0.001 |
| Squid-sashimi | 1.72 (1.23 - 2.81) | 0.003 | 0.003 | 0.98 (0.72 - 1.35) | 0.888 | 0.888 | 1.20 (0.93 - 1.55) | 0.161 | 0.161 | 0.81 (0.49 - 1.32) | 0.396 | 0.397 |
| Easy-to-chew food | | | | | | | | | | | | |
| Steamed rice | 4.93 (2.35 - 10.30) | <0.001 | <0.001 | - | - |  | 4.93 (2.36 - 10.30) | <0.001 | <0.001 | 1.28 (1.01 - 1.61) | 0.038 | 0.038 |
| Tuna sashimi | 1.55 (0.57 - 4.18) | 0.390 | 0.390 | 0.70 (0.40 - 1.23) | 0.212 | 0.212 | 0.81 (0.49 - 1.32) | 0.397 | 0.397 | 1.27 (1.01 - 1.58) | 0.038 | 0.039 |
| Grilled eel | 1.04 (0.57 - 1.92) | 0.893 | 0.893 | 0.79 (0.54 - 1.15) | 0.219 | 0.219 | 0.85 (0.61 - 1.17) | 0.315 | 0.315 | 1.32(1.06-1.65) | 0.013 | 0.013 |

Among the 15 foods investigated in this study, 10 had statistically significant hazard ratios for mortality in men. In contrast, no food had a statistically significant hazard ratio in women.
