# Supplementary material for: Effects of self-assessed chewing ability, tooth loss and serum albumin on mortality in 80-year-old individuals: a 20-year follow-up study
Source: BMC Oral Health. 2020 Apr 21;20:122. doi: 10.1186/s12903-020-01113-7 (PMC7175538; doi:10.1186/s12903-020-01113-7)
Supplement: Supplementary file 4 — Additional file 4: Table S4. Item parameter estimates for the 3-parameter logistic model. [file 12903_2020_1113_MOESM4_ESM.docx]

**Table S4 Item parameter estimates for the 3-parameter logistic model**

|  | Item discrimination | Item difficulty | Asymptotes |
| --- | --- | --- | --- |
| Very hard-to-chew food | | | |
| Peanuts | 2.301 | -0.680 | 0.053 |
| Yellow pickled radish | 2.604 | -0.641 | 0.107 |
| Hard rice crackers | 2.274 | -0.854 | <0.001 |
| Moderately hard-to-chew food | | | |
| French bread | 1.720 | 0.003 | 0.128 |
| Beefsteak | 1.689 | -0.418 | <0.001 |
| Octopus in vinegar | 2.790 | -0.183 | 0.041 |
| Pickled shallots | 1.938 | -1.123 | <0.001 |
| Dried scallops | 2.903 | 0.139 | <0.001 |
| Dried cuttlefish | 3.721 | 0.180 | 0.014 |
| Slightly hard-to-chew food | | | |
| Konnyaku-jelly | 2.484 | -0.87 | 0.092 |
| Tubular roll of boiled fish paste | 3.582 | -1.812 | 0.002 |
| Squid-sashimi | 2.366 | -2.007 | <0.001 |
| Easy-to-chew food | | | |
| Steamed rice | 1.989 | -2.14 | 0.774 |
| Tuna sashimi | 0.825 | -3.865 | 0.016 |
| Grilled eel | 1.253 | -1.456 | 0.398 |
