# Supplementary material for: Effects of self-assessed chewing ability, tooth loss and serum albumin on mortality in 80-year-old individuals: a 20-year follow-up study
Source: BMC Oral Health. 2020 Apr 21;20:122. doi: 10.1186/s12903-020-01113-7 (PMC7175538; doi:10.1186/s12903-020-01113-7)
Supplement: Supplementary file 5 — Additional file 5: Table S5. Effect of the use of dentures on the mortality of edentulous subjects adjusted by serum albumin levels. [file 12903_2020_1113_MOESM5_ESM.docx]

**Table S5 Effect of the use of dentures on the mortality of edentulous subjects adjusted by serum albumin levels**

|  | **Men** | | | **Women** | | | **Total** | | | **Strata model (Strata by sex)** | | |
| --- | --- | --- | --- | --- | --- | --- | --- | --- | --- | --- | --- | --- |
|  | Hazard Ratio (95% CI) | P-value | Model fit | Hazard Ratio (95% CI) | P-value | Model fit | Hazard Ratio (95% CI) | P-value | Model fit | Hazard Ratio (95% CI) | P-value | Model fit |
| Denture(-) / Denture(+) | 3.01 (1.18 - 7.69) | 0.022 | <0.001 | 2.38 (0.58 - 10.00) | 0.228 | 0.030 | 0.96 (0.45 - 2.02) | 0.955 | 0.207 | 0.86 (0.40 - 1.82) | 0.691 | <0.001 |
| Serum albumin (mg/dL) | 2.72 (0.96 - 7.75) | 0.059 |  | 10.42 (2.51 - 43.5) | 0.001 |  | 0.49 (0.32 - 0.73) | 0.001 |  | 0.47 (0.31 - 0.70) | <0.001 |  |
