# Supplementary material for: Effects of self-assessed chewing ability, tooth loss and serum albumin on mortality in 80-year-old individuals: a 20-year follow-up study
Source: BMC Oral Health. 2020 Apr 21;20:122. doi: 10.1186/s12903-020-01113-7 (PMC7175538; doi:10.1186/s12903-020-01113-7)
Supplement: Supplementary file 6 — Additional file 6: Figure S1. Item response curves for the 15 different types of foods. Figure S2 Survival curves for serum albumin, edentulous/dentulous status and masticatory dysfunction. [file 12903_2020_1113_MOESM6_ESM.pptx]

## Slide 1
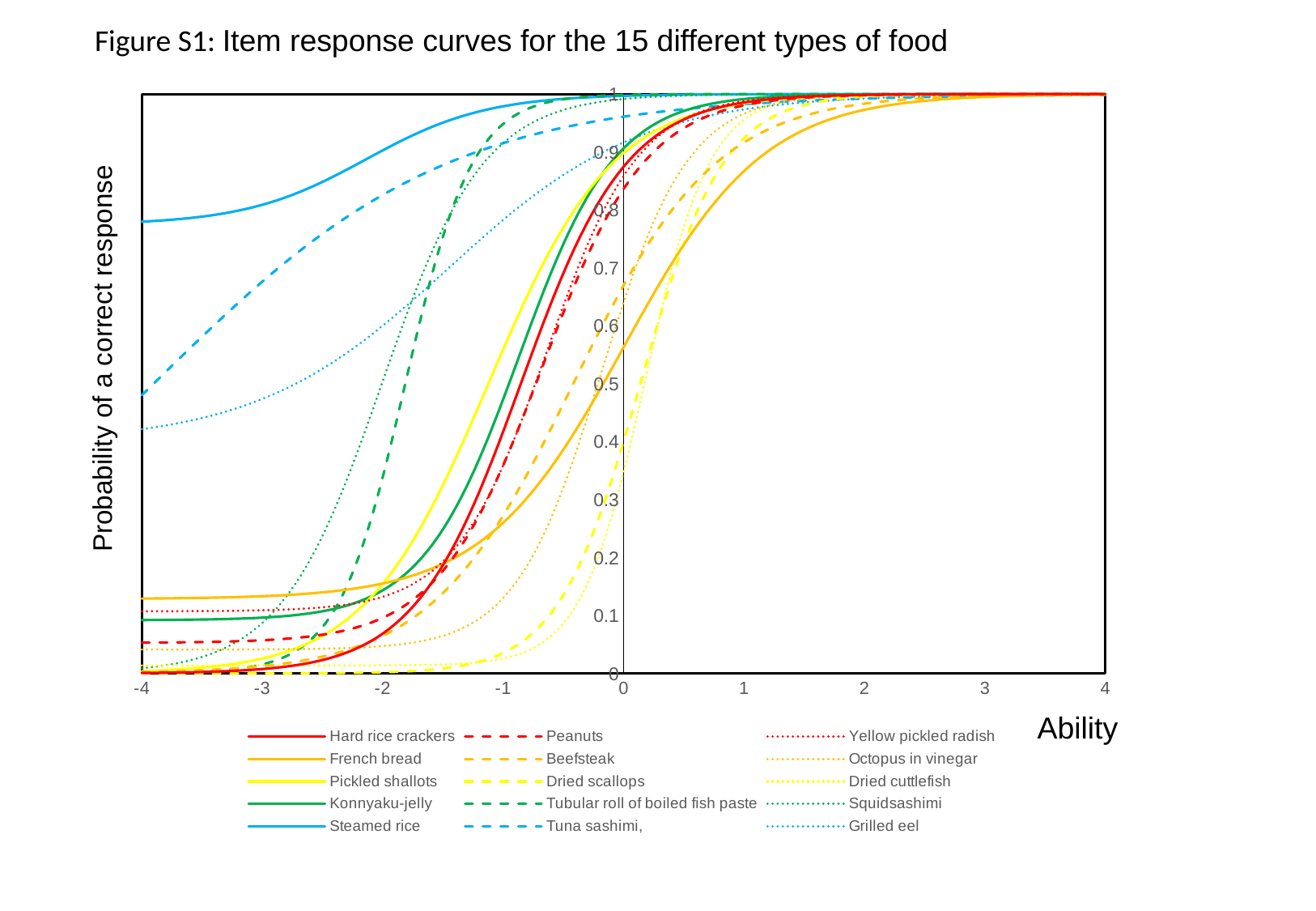

Figure S1: Item response curves for the 15 different types of food
### Chart
| Category | Hard rice crackers | Peanuts | Yellow pickled radish | French bread | Beefsteak | Octopus in vinegar | Pickled shallots | Dried scallops | Dried cuttlefish | Konnyaku-jelly | Tubular roll of boiled fish paste | Squidsashimi | Steamed rice | Tuna sashimi, | Grilled eel |
|---|---|---|---|---|---|---|---|---|---|---|---|---|---|---|---|Probability of a correct response
Ability

## Slide 2
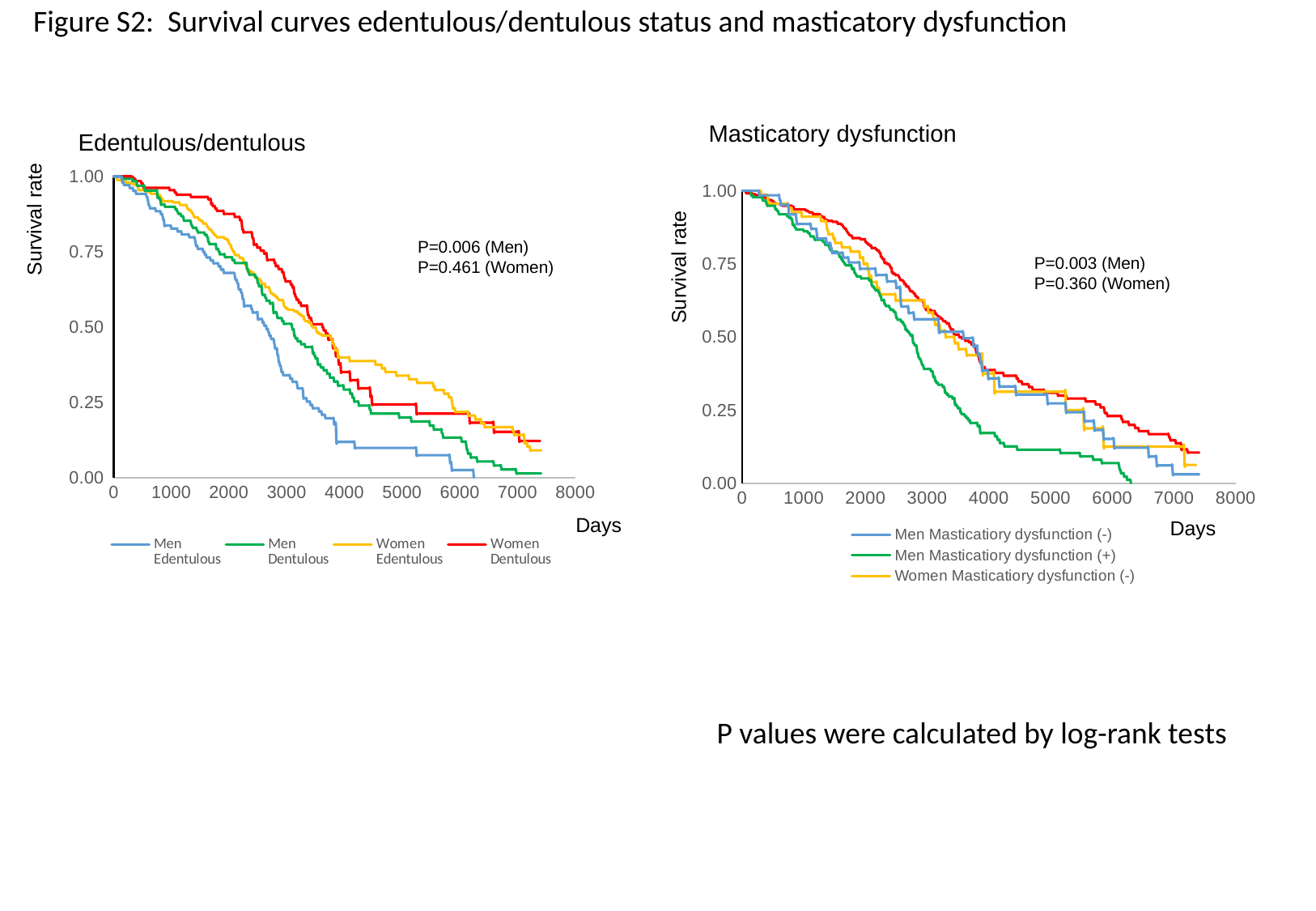

Figure S2: Survival curves edentulous/dentulous status and masticatory dysfunction
Masticatory dysfunction
Edentulous/dentulous
### Chart
| Category | Men
Edentulous | Men
Dentulous | Women
Edentulous | Women
Dentulous |
|---|---|---|---|---|
### Chart
| Category | Men Masticatiory dysfunction (-) | Men Masticatiory dysfunction (+) | Women Masticatiory dysfunction (-) | Women Masticatiory dysfunction (+) |
|---|---|---|---|---|Survival rate
P=0.006 (Men)
P=0.461 (Women)
P=0.003 (Men)
P=0.360 (Women)
Survival rate
Days
Days
P values were calculated by log-rank tests
